# Identification of biomarkers for radiation-induced acute intestinal symptoms (RIAISs) in cervical cancer patients by serum protein profiling

**DOI:** 10.1093/jrr/rru081

**Published:** 2014-09-24

**Authors:** Yanlan Chai, Juan Wang, Ying Gao, Tao Wang, Fan Shi, Jin Su, Yunyi Yang, Xi Zhou, Liping Song, Zi Liu

**Affiliations:** 1 Department of Radiotherapy Oncology, First Affiliated Hospital of Xi'an Jiaotong University, Xi'an 710061, P. R. China; 2 Renmin Hospital, Hubei University of Medicine, Hubei 442000, P. R. China

**Keywords:** biomarker identification, radiation-induced acute intestinal symptom, cervical cancer, protein profiling, SELDI-TOF MS

## Abstract

Radiation-induced acute intestinal symptoms (RIAISs) are the most frequent complication of radiotherapy that causes great pain and limits the treatment efficacy. The aim of this study was to identify serum biomarkers of RIAISs in cervical cancer patients by surface-enhanced laser desorption/ionization time-of-flight mass spectrometry (SELDI-TOF MS). Serum samples were collected from 66 cervical cancer patients prior to pelvic radiotherapy. In our study, RIAISs occurred in 11 patients. An additional 11 patients without RIAISs were selected as controls, whose age, stage, histological type and treatment methods were matched to RIAISs patients. The 22 sera were subsequently analyzed by SELDI-TOF MS, and the resulting protein profiles were evaluated to identify biomarkers using appropriate bioinformatics tools. Comparing the protein profiles of serum samples from the RIAIS group and the control group, it was found that 22 protein peaks were significantly different (*P* < 0.05), and six of these peaks with mass-to-charge (m/z) ratios of 7514.9, 4603.94, 6887.41, 2769.21, 3839.72 and 4215.7 were successfully identified. A decision tree model of biomarkers was constructed based on three biomarkers (m/z 1270.88, 1503.23 and 7514.90), which separated RIAIS-affected patients from the control group with an accuracy of 81%. This study suggests that serum proteomic analysis by SELDI-TOF MS can identify cervical cancer patients that are susceptible to RIAISs prior to pelvic radiotherapy.

## INTRODUCTION

Radiotherapy is often used as a definitive therapy for the management of patients with locally advanced cervical cancer or patients who are poor candidates for surgery. It is also used as an adjuvant therapy in patients who have undergone radical hysterectomy and have one or more pathologic risk factors, such as positive nodes and/or positive surgical margins [1]. However, radiotherapy remains dose-limited because of the surrounding normal tissue tolerances. For cervical cancer patients, the intestine is a major dose-limiting organ during pelvic radiotherapy [2]. Radiation-induced acute intestinal symptoms (RIAISs) occur in 50–75% of cervical cancer patients, which may lead to dose reduction, treatment interruptions, increased healthcare costs, and impaired quality of life [3]. The symptoms include abdominal pain, diarrhea, and tenesmus, and often resolve in 2–6 months [3]. Molecular markers of individual response to radiation would greatly improve planning and monitoring of radiotherapy.

The proteome represents the entire set of proteins and peptides in a biological sample, and proteomics is a useful tool for monitoring disease presence, progression, and the response to therapy [4]. Among various techniques conventionally used for proteomic profiling, surface-enhanced laser desorption/ionization time-of-flight mass spectrometry (SELDI-TOF MS) represents a high-throughput analysis approach that has overcome many of the limitations of both 2D electrophoresis and matrix-assisted laser desorption/ionization time-of-flight mass spectrometry (MALDI-TOF MS) [5]. Using chromatographic surfaces to retain proteins and peptides on ProteinChip arrays based on their physicochemical properties, the platform performs direct analysis via TOF MS [6]. The result is a mass spectrum comprised of the mass-to-charge ratio (m/z) and intensities of the bound peptide/protein [7]. Combined with bioinformatics approaches, SELDI-TOF MS is valuable in establishing protein expression profiles and in discovering new biomarkers in biological fluids (such as plasma, serum, tissue and urine) with high sensitivity and specificity [6, 8]. SELDI-TOF MS has been successfully applied for the screening of biomarkers in various types of tumors, such as gastric [9], colon [7], breast [10], lung [11] and ovarian cancers [12].

Some studies have explored biomarkers that predict and monitor radiation-related side effects in prostate cancer [13, 14]. However, reports about biomarkers of radiation-related damages in cervical cancer are rare. Herein we describe a proteomics study using SELDI-TOF MS combined with bioinformatic analysis to identify biomarkers of RIAISs in cervical cancer patients.

## MATERIALS AND METHODS

### Patient recruitment

A total of 66 patients with cervical cancer who had adequate performance status were recruited between June and August 2012. The patients had no history of colitis, diabetes or pelvic inflammatory disease. The study protocol was approved by the Ethics Committee of the First Affiliated Hospital of Xi'an Jiaotong University, and informed consents were obtained from all participants prior to sample collection.

### Sample collection and storage

After fasting and avoiding alcohol and medicine for 12 h, the participants had blood samples taken 1–3 days before radiotherapy. About 1 ml of serum was obtained after centrifugation at 3000 rpm for 10 min, and samples were stored at −80°C until analysis. All the chemicals that are mentioned in the following sections were purchased from Sigma–Aldrich (St Louis, MO, USA), unless stated otherwise.

### Specimen preparation

Serum samples were thawed on ice and centrifuged at 10 000 rpm for 2 min at 4°C, and supernatants were collected. Samples that were either hemolyzed or visually lipemic were excluded from analysis. Samples of 5 µl of the supernatants were diluted with 20 µl of U9 buffer (9M urea, 2% CHAPS, 1% DTT and 50 mM Tris-HCl, pH 9.0), and vortexed at 4°C for 30 min. Finally, 175 µl of binding buffer (50mM NaAC, pH 4.0) was added to samples, which resulted in a final dilution of 40-fold.

### ProteinChip processing

The SELDI ProteinChip arrays of weak cation exchange (CM10) from Ciphergen Biosystems (Fremont, CA) were assembled in a bioprocessor, which was washed twice with 200 ml of binding buffer for each well with gentle shaking for 5 min, keeping the surface of each spot wet. Then, 100 µl of diluted serum sample was added to each well of the bioprocessor and shaken at 500 rpm for 1 h at 4°C. After discarding unbound samples, each well was washed twice with 200 µl of binding buffer, followed by washing with 200 µl of HPLC water. After all spots were air-dried, 0.5 µl of sinapinic acid solution (a semi-saturated solution of sinapinic acid in 50% acetonitrile and 0.5% trifluoroacetic acid) was added to each spot twice, allowing the surface to dry between each application.

### SELDI-TOF MS and data processing

All chips were analyzed in a single Protein Biological System IIc SELDI-TOF MS (Ciphergen Biosystems, Fremont, CA; Serial No. 3B480TC). Each spot was scanned by a laser with the intensity of 195, and peptides were detected with a sensitivity of 7.

The authors limited the peptide detection range from 2 to 10 kDa. The instrument was calibrated by the all-in-one peptide molecular mass standard (Ciphergen Biosystems, Fremont, CA). Peaks were detected and grouped after total ion current normalization and baseline subtraction by using Ciphergen Biosystems ProteinChip Software v.3.2.1 with the signal-to-noise ratio >5 for the first pass and >2 for the second pass, a 0.1% cluster mass window, and a requirement for peaks to be present in >5% of the spectra. Data from duplicate spectra were combined prior to further analysis.

### Statistical analysis

The statistical significance of peak height difference between samples from RAIS participants and control participants was calculated by the Student's *t*-test. Hierarchical clustering of significantly changed peaks was conducted with MATLAB v.7.5 (MathWorks, Natick, MA, USA) by using default parameters (Euclidean distance metric and average linkage method). All statistical analyses were performed with a predetermined significance level of *α* = 0.05.

### Decision tree

The decision tree was constructed by the Package weka.classifiers.trees.J48 (C4.5 java version). Splitting decisions were based on normalized intensity levels of peaks. The decision tree split the data into two nodes using one rule at a time in the form of peak intensity, and the splitting process was continued until terminal nodes were created. The accuracy of the generated decision tree was estimated through a process of 4-fold cross-validation.

### Biomarker identification

First, all proteins or peptide sequences in the UniProtKB/Swiss-Prot biological database (http://www.ebi.ac.uk/swissprot/, 10 November 2013, date last accessed) [15, 16] were converted to theoretical m/z values. Then, each m/z value of peaks detected by SELDI-TOF MS was compared with all theoretical m/z values repeatedly. The significance of the result was evaluated by the Link-test. The *P*-value was assigned to 0.05 by binomial test using default parameters with δ = 0.01 [17].

### Biomarker detection by ELISA

To test intensities of candidate biomarkers, serum samples of another 23 RIAIS participants and 10 matched control participants were collected prior to pelvic radiation. Candidate biomarkers were detected by enzyme-linked immunosorbent assay (ELISA; AssayMax Human ProAlbumin ELISA Kit, BioCat GmbH, Heidelberg, Germany). Tests were performed according to the manufacturer's instructions. Duplicate determinations were carried out for all samples.

## RESULTS

### Patient treatment and outcome

Patient treatment was performed according to the National Comprehensive Cancer Network Guidelines [1]. A total of 34 patients underwent radical hysterectomy followed by external-beam radiotherapy (EBRT) (45–50.4 Gy) in 25–28 fractions, while 32 patients received radical radiotherapy with 50.4 Gy EBRT in 28 fractions followed by intracavitary brachytherapy (20–25 Gy). The RIAISs occurrence was assessed on a daily basis by a single observer, and was graded according to the National Cancer Institute Common Toxicity Criteria version 2.0 [18].

Overall, radiotherapy was well tolerated, with no recorded Grade 4 or 5 adverse events. However, RIAISs occurred in 11 patients (16.7%), of which four and seven patients experienced Grade 2 and 3 proctitis, respectively. The main symptoms included abdominal pain, diarrhea and tenesmus. All cases were successfully managed by conservative treatment.

For the control group, 11 patients without RIAISs were selected, whose age, stage, histological type, and treatment methods were matched to the RIAISs patients. A summary of patient characteristics is presented in Table 1.

**Table 1.** Patient characteristics for the RIAIS group and the control group

Characteristics	RIAIS group	Control group
No. of women	11	11
Age (years)		
Median/mean	54/51.9	55/54.2
Range	30–67	37–65
FIGO stage		
I	2	2
II	6	7
III	2	2
IV	1	0
Histological type		
SqCa	11	11
Therapy		
Radical hysterectomy and adjuvant radiotherapy	7	7
Radical radiotherapy	4	4

### Comparison of protein spectra in serum samples from RIAISs patients with those from control patients

**Fig. 1.**
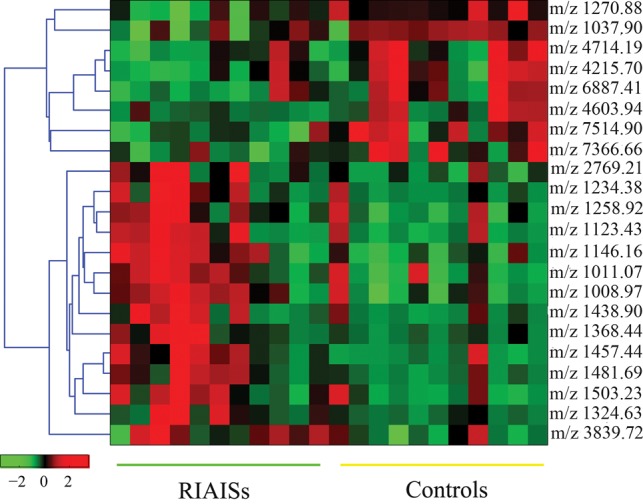
Heat map of SELDI peak intensities in serum of RIAISs patients and control patients. Results are ordered (left to right) for Patients 1–11 (RIAISs) and Patients 1–11 (controls). Peak intensities of the SELDI spectra are shown on a sliding scale from green, indicating low intensity, to red, indicating high intensity.

A total of 253 cluster peaks was detected. Student's *t*-test was carried out to identify peaks with statistically significant differences between the RIAISs group and the control group. We found that 22 peaks differed significantly between these groups of patients (*P* < 0.05) (Table 2). The results are summarized as a heat map in Fig. 1, which shows that 8 m/z peaks were lower and 14 were higher in the RIAISs group compared with the control group.

**Table 2.** SELDI protein peak intensities in serum with statistically significant differences (*P* < 0.05, from paired *t*-tests) between the RIAIS group patients (*n* = 11) and the control group patients (*n* = 11)

Protein peaks (m/z)	Intensity (mean)		*P*-value
	RIAISs	controls	
1270.88*^a^*	−0.65372	0.263178	0.003422
1146.16	3.706879	1.55921	0.004195
7514.90*^a^*	0.215618	1.051347	0.004503
1008.97	13.77295	8.41053	0.006082
1457.44	1.379338	0.311297	0.009984
1481.69	2.52233	0.629674	0.019357
1368.44	1.412335	0.362662	0.023833
4603.94*^a^*	0.448671	4.382222	0.024781
6887.41*^a^*	1.383407	3.837666	0.030072
2769.21	1.588135	0.656592	0.031762
1503.23	2.326273	0.90928	0.034871
1123.43	2.425003	0.945949	0.035298
3839.72	0.171704	−0.2036	0.035357
1324.63	1.279412	0.269564	0.036842
1011.07	8.129393	4.865236	0.039046
4215.70*^a^*	1.763202	3.924723	0.039063
4714.19*^a^*	0.934337	2.081019	0.039941
1438.90	1.409347	0.41523	0.041148
1258.92	4.244041	2.378696	0.04507
1234.38	6.594352	1.942057	0.036502
7366.66*^a^*	0.089043	0.764678	0.017394
1037.90*^a^*	−0.99219	−0.35862	0.020794

Peaks labeled with ‘*^a^*’ were lower in the RIAIS group than in the control group.

### Quality control and reproducibility

The reproducibility of each SELDI ProteinChip assay spectra, that is the variability of mass and intensity from array to array on a single CM10 chip (intra-assay) and between chips (inter-assay), was determined by using a serum sample from a healthy volunteer. Both the coefficient of variation (CV) for intensity and m/z were calculated based on a duplicate sample testing. The intrachip and interchip CV for intensity were <5%. Both the intrachip and interchip CV for m/z were <0.05%, which confirmed the high reproducibility of spectra with the SELDI-TOF MS.

### Decision tree

**Fig. 2.**
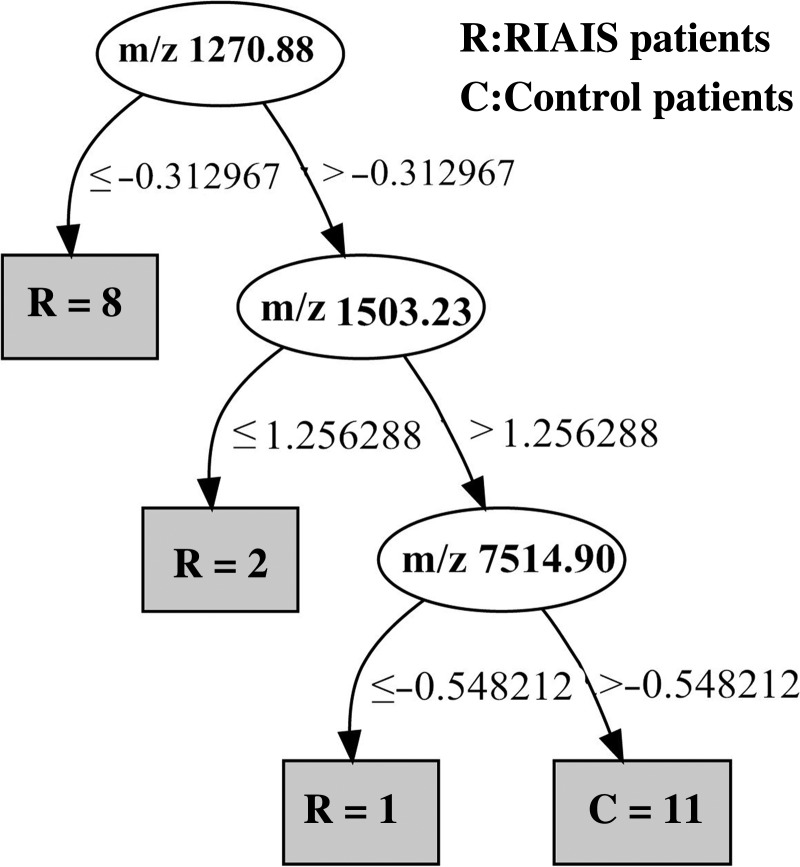
Decision tree. Classification of serum samples from RIAISs patients (*n* = 11) and control patients (*n* = 11) by using a decision tree algorithm. Cut-off points for m/z 1270.88, 1503.23 and 7514.90 were −0.312967, 1.256288 and −0.548212, respectively.

In an attempt to build a diagnostic model for RIAISs, the 22 peaks with statistically significant differences between the RIAISs group and the control group were used to construct a decision tree. The Package weka.classifiers.trees.J48 (C4.5 java version) identified a series of decision tree models that were constructed with one or more protein peaks with varying classification accuracy. The best decision tree with the highest classification accuracy was constructed using peaks with m/z 1270.88, 1503.23 and 7514.90 (Fig. 2). The selected decision tree is simple and straightforward, and used three splitters and classified three terminal nodes. Cut-off points for m/z 1270.88, 1503.23 and 7514.90 were −0.312967, 1.256288 and −0.548212, respectively. Left branch nodes after the splitter depict the cases of peak intensity equal to or lower than the cut-off point, while right branch nodes depict cases greater than the cut-off point. The accuracy of the decision tree after 4-fold cross-validation was 81%.

### Biomarker identification

Six biomarkers for RIAISs were successfully identified by comparing m/z values with theoretical m/z values from the UniproKb/Swiss-Prot database. The peaks included 7514.9, 4603.94, 6887.41, 2769.21, 3839.72 and 4215.7, which were identified as the osteopontin (OPN) fragment, the neurosecretory protein VGF fragment, transthyretin (TTR), hepcidin, the neurosecretory protein VGF fragment, and the plasma protease C1-inhibitor (C1-INH) fragment, respectively. Two peptides with m/z values of 4603.94 and 3839.72 were derived from the same protein (neurosecretory proteins VGF). The results were summarized in Table 3.

**Table 3.** Identified biomarkers for RIAIS group patients

m/z	Predicted protein	Theoretical m/z values	Gene name
7514.9	OPN fragment	7658.19	*SPP1*
4603.94^a^	Neurosecretory protein VGF fragment	4823.5	*VGF*
6887.41	TTR	6880	*TTR*
2769.21	Hepcidin	2797.41	*HAMP*
3839.72^a^	Neurosecretory protein VGF fragment	3688.03	*VGF*
4215.7	Plasma protease C1-inhibitor fragment	4152.87	*SERPING1*

^a^The two peptides with m/z values of 4603.94 and 3839.72 are derived from the same protein neurosecretory protein, VGF.

### ELISA

**Fig. 3.**
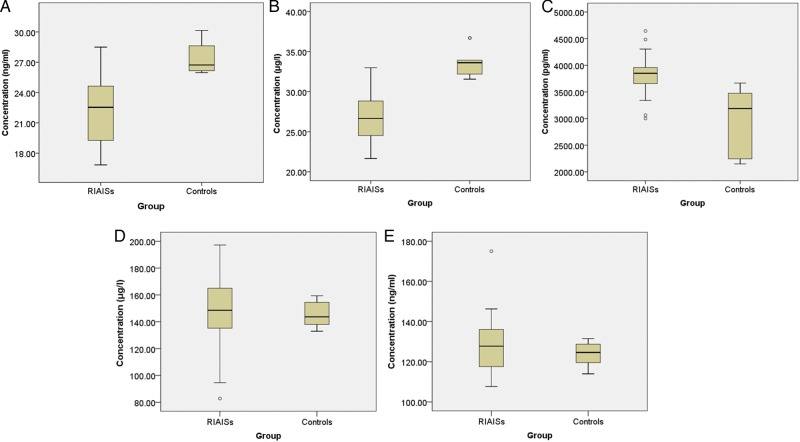
Box plot of serum concentration of TTR (**A**), OPN (**B**), neurosecretory protein VGF (**C**), hepcidin (**D**) and C1-INH (**E**) measured by ELISA. The RIAISs group (*n* = 23) showed significantly lower levels of TTR (22.112 vs 27.528 ng/ml; *P* = 0.001) and OPN (26.650 vs 33.608 μg/ml; *P* = 0.03), and significantly higher levels of neurosecretory protein VGF (3824.244 vs 2944.890 pg/ml; *P* = 0.002) than control patients (*n* = 10), while concentrations of hepcidin and C1-INH were not significantly different between RIAIS patients and control patients (*P* > 0.05). The thick line in the boxes indicates the median value of intensities. Upper and lower areas of boxes indicated ranges of the value from 25–75%. Small circles indicate outliers.

Serum levels of OPN, TTR, the neurosecretory protein VGF, hepcidin, and plasma protease C1-inhibitor were detected in another 23 RIAISs patients and 10 matched control individuals. The RIAISs group showed significantly lower levels of TTR (22.112 vs 27.528 ng/ml; *P* = 0.001), OPN (26.650 vs 33.608 μg/ml; *P* = 0.03), and a significantly higher level of neurosecretory protein VGF (3824.244 vs 2944.890 pg/ml; *P* = 0.002). In contrast, concentrations of hepcidin and C1-INH were not significantly different between RIAISs and controls (*P* > 0.05) (Fig. 3).

## DISCUSSION

Pelvic radiotherapy, a standard treatment for cervical cancer patients, is usually accompanied by either acute or late complications. Acute radiation injury of the intestine induces mucosal inflammation and damage to endothelial cells and microvessels [19], and triggers apoptosis of intestinal crypt cells [20]. Increased damaged vessels chemotaxis and thrombogenesis are the main mechanisms contributing to radiation enteropathy [19, 20]. These events lead to mucosal dysfunction and ultimately result in RIAISs, which are characterized by abdominal pain, diarrhea, tenesmus, and even bloody stools. It is recognized that factors such as age, smoking, total radiation dose, and fractionation schedule contribute to the severity ofo RIAISs [21, 22]; however, there are few studies analyzing molecular biomarkers and simultaneously predicting RIAISs in cervical cancer patients.

In this study, we combined the SELDI ProteinChip technology and artificial intelligence algorithms to identify candidate biomarkers that would be able to predict RIAISs in cervical cancer patients. We found that the serum levels of eight peaks were lower and the serum levels of 14 peaks were higher in the RIAISs group compared with the control group. Based on these 22 peaks, a decision tree was constructed that identifies patients susceptible to RIAISs with significant comparable accuracy. From these 22 peaks, six biomarkers (m/z 7514.9, 4603.94, 6887.41, 2769.21, 3839.72 and 4215.7) were successfully identified as the OPN fragment, the neurosecretory protein VGF fragment (two peaks), TTR, hepcidin and the C1-INH fragment. The OPN fragment, TTR, and the C1-INH fragment were downregulated, while hepcidin and the two kinds of neurosecretory protein VGF fragments were upregulated in RIAISs patients.

OPN is a multifunctional matricellular cytokine present in most tissues and body fluids. It has an important modulatory role in innate immunity by stimulating various signal transduction pathways [23–25], which is reflected in its mucosal protective function [23]. Furthermore, OPN has been shown to modulate the radiosensitivity of cancer cells. Hahnel *et al.* [26] reported that OPN deficiency improves radiobiological effects in MDA-MB-231 cells, and Yang *et al.* [27] suggested that suppression of OPN gene expression enhances radiosensitivity in breast cancer cells. In this study, we found that OPN was downregulated in RIAS patients, suggesting that OPN deficiency enhances the radiosensitivity of the intestinal mucosa and contributes to the development of RIAISs.

Neurosecretory protein VGF is a secreted polypeptide that is expressed by neurons and several endocrine and neuroendocrine tissues [28, 29]. TTR is a thyroid hormone-binding protein that transports thyroxine from the bloodstream to the brain [30]. Decreased serum TTR levels could imply abnormalities in transport and dysfunction of thyroxin in the brain. Huang *et al.* [31] found increased levels of a neurosecretory protein VGF-derived peptide (VGF23-62 fragment) and decreased TTR protein concentrations in the cerebrospinal fluid of patients with the initial prodromal states of psychosis and schizophrenia. The 4.82-kDa neurosecretory protein VGF peptide was found to be downregulated in Alzheimer's disease [32], while the 3.69-kDa neurosecretory protein VGF peptide was reported to be decreased in frontotemporal dementia [33]. Asano *et al.* [34] identified the 4.82-kDa neurosecretory protein VGF in the cerebrospinal fluid as a novel biomarker for encephalopathy by using SELDI-TOF MS. All these studies highlight the importance of neurosecretory protein VGF and TTR in neurological/psychiatric disorders. Therefore, we infer that the RIAISs occurrence is related to the mental status of patients before pelvic radiotherapy. Future studies are required to explore possible functions of TTR and neurosecretory protein VGF peptides in intestinal homeostasis.

Hepcidin is an acute phase protein that also plays a key role in the regulation of iron intestinal absorption [35, 36]. Furthermore, Christiansen *et al.* [14] examined serum and urine hepcidin levels in 18 patients undergoing radiotherapy for prostate cancer and found that hepcidin levels were significantly increased during radiotherapy in prostate cancer patients who develop acute proctitis. Therefore, hepcidin may serve as a predictive biomarker and a therapeutic target in radiation-induced proctitis. However, larger clinical studies are needed to explore a potential role of hepcidin in RIAISs.

C1-INH, produced mainly in the liver, is the main inhibitor of the early activation steps of the classical complement pathway. In the presence of C1-INH deficiency, the classical complement pathway can be inappropriately or excessively activated. In this study, the serum level of C1-INH was downregulated in RIAISs patients, despite no significant difference in ELISA results. Therefore, we inferred that RIAISs may be associated with C1-INH deficiency and excessive activation of the classical complement pathway.

However, the peaks with m/z 1270.88 and 1503.23 in the decision tree are still unknown, and are very likely biologically active and not merely incidental fragments. Further studies are needed to explore their natures, structures and functions.

Based on the expression of these proteins, we infer that RIAISs may be related to intestinal barrier dysfunction, chronic intestinal inflammation, C1-INH deficiency and perhaps the psychological status of patients before radiotherapy. Further investigations are also required to explore the roles of these proteins in RIAISs and the etiology and pathology of this disease.

This study revealed that patients susceptible to RIAISs can be identified prior to pelvic radiotherapy. Individualized treatment programs could be designed for these patients, and appropriate protective measures, such as more advanced radiation technology to guarantee radiation dose distribution with high precision, could be taken. In addition, the radiation dose could be reduced and the prophylactic administration of antidiarrheal drugs and other supportive treatment could be prescribed for patients at risk for RIAISs. These measures could overcome the discontinuation of pelvic radiotherapy or at least alleviate RIAISs. In addition, differently expressed proteins that were identified may represent potential therapeutic targets for RIAISs; however, it needs to be established whether targeting these proteins would be beneficial or deleterious. In addition, identification of the unknown peaks also needs further effort.

In conclusion, we showed that SELDI protein profiling of serum can identify cervical cancer patients susceptible to RIAISs with relatively high sensitivity and specificity. Therefore, SELDI-TOF MS may be used as a promising tool for screening and identification of patients susceptible to RIAISs.

## FUNDING

This study was financially supported by the National Natural Science Foundation of China (No. 81071838), the Department of Health Key Program of Shaanxi Province (No.2010A02), and the First Affiliated Hospital of Xi'an Jiaotong University (No. 2010YK3).
